# Impact of Vestibular and Balance Rehabilitation Therapy Along With Conventional Physiotherapy in a Case of Vestibular Schwannoma With CP Angle Tumor: A Case Report

**DOI:** 10.7759/cureus.45224

**Published:** 2023-09-14

**Authors:** Vaishnavi B Warutkar, Snehal Samal, Prishita Koul

**Affiliations:** 1 Neuro Physiotherapy, Ravi Nair Physiotherapy College, Wardha, IND

**Keywords:** facial pnf, case report, vestibulocochlear nerve, physiotherapy, vestibular rehabilitation, facial nerve

## Abstract

Vestibular Schwannoma forms in Schwann cells which cover the vestibular nerve, often in the internal auditory canal. Symptoms were likely present before the excision of the tumor. After the excision of the tumor, symptoms may include, hearing defects, tinnitus, facial muscle abnormalities, headache, and balance impairments. This case report is of a female patient with vestibular schwannoma, who had a few above-mentioned symptoms after the surgery. Her physiotherapy protocol included Vestibular and Balance Rehabilitation Therapy along with Conventional Physiotherapy. Also, facial proprioceptive neuromuscular facilitation (PNF) was a part of her treatment. The outcome measures used to rule out the results were the motion sensitivity quotient (MSQ) and the Berg balance scale (BBS). Improvement of symptoms is likely related to the excision of the tumor and the therapy program. The person's balance and coordination improved as a result of the rehabilitation protocol, and she was able to do her regular tasks with minimal assistance. Her standard of living was also enhanced.

## Introduction

One in 100,000 people will develop a benign cerebral tumor called a schwannoma. Schwannoma often manifests between the ages of 40 and 60 [1]. 80-90% of these instances come from the vestibular nerve [2]. A benign tumor called a vestibular schwannoma (also known as an acoustic neuroma) develops from the Schwann cells which cover the vestibular nerve, often in the internal auditory canal [3]. Continuous growth might eventually result in intracranial pressure, brain stem compression, hydrocephalus, and death [4]. The significant indications for microsurgery include massive, expanding tumors, tumors with bothersome symptomatology (vertigo, tinnitus), and when the patient prefers it [5]. Various symptoms are seen after vestibular schwannoma excision, including hearing problems, tinnitus, facial nerve abnormalities, headaches, and balance issues [6]. Following acoustic neuroma surgery, dysequilibrium is frequently noted in the acute period, but they typically get better over time [7]. Normally, this procedure takes a few weeks to a few months. Some patients never complete it, which results in balance issues that significantly impair their quality of life and cause considerable challenges in everyday tasks [4].

Facial nerve (FN) palsy is still a common post-vestibular schwannoma surgery consequence, considering the most recent advancements in surgical instruments and intra-operative monitoring. The size of the tumor can put its anatomic-functional viability at risk following removal depending on the existence and severity of FN deficiency, although this is not always the case. In general, a postoperative FN deficit following vestibular schwannoma removal is less frequent when the nerve is physically intact, but, unfortunately, it is also conceivable after the excision of a tiny, intracanalicular vestibular schwannoma [8].

Subsequently, according to numerous controlled, prospective trials conducted on these patients, a vestibular training program improves postural stability and subjectively alleviates imbalance in individuals with chronic vestibular loss [9]. This case report describes a patient with vestibular schwannoma who has undergone surgery for the excision of that tumor. A comprehensive rehabilitation plan was necessary to reduce her difficulties and enhance her quality of life. Her treatment protocol included vestibular rehabilitation along with conventional physiotherapy that involved facial proprioceptive neuromuscular facilitation (PNF).

## Case presentation

Patient information

A 51-year-old female patient was brought to our inpatient ward with complaints of headaches, blurring of vision in both eyes, imbalance while walking, and dizziness for one month. She didn't use any assistive device while walking. At our hospital, an MRI and CT scan were done. It revealed a heterogeneously enhancing extra-axial, lobulated mass in the left cerebellopontine angle cistern involving the seventh and eighth nerve complex.

Clinical findings

**Figure 1:**
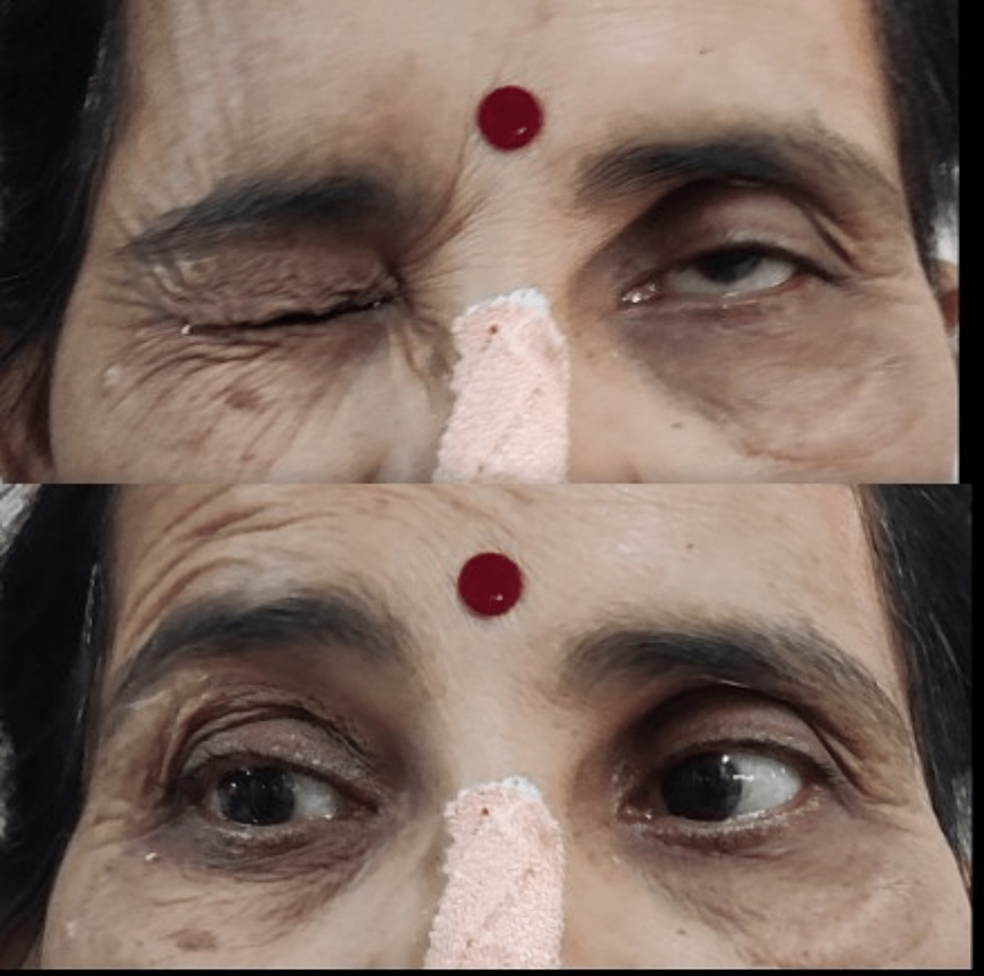
Facial muscles assessment.

**Figure 2:**
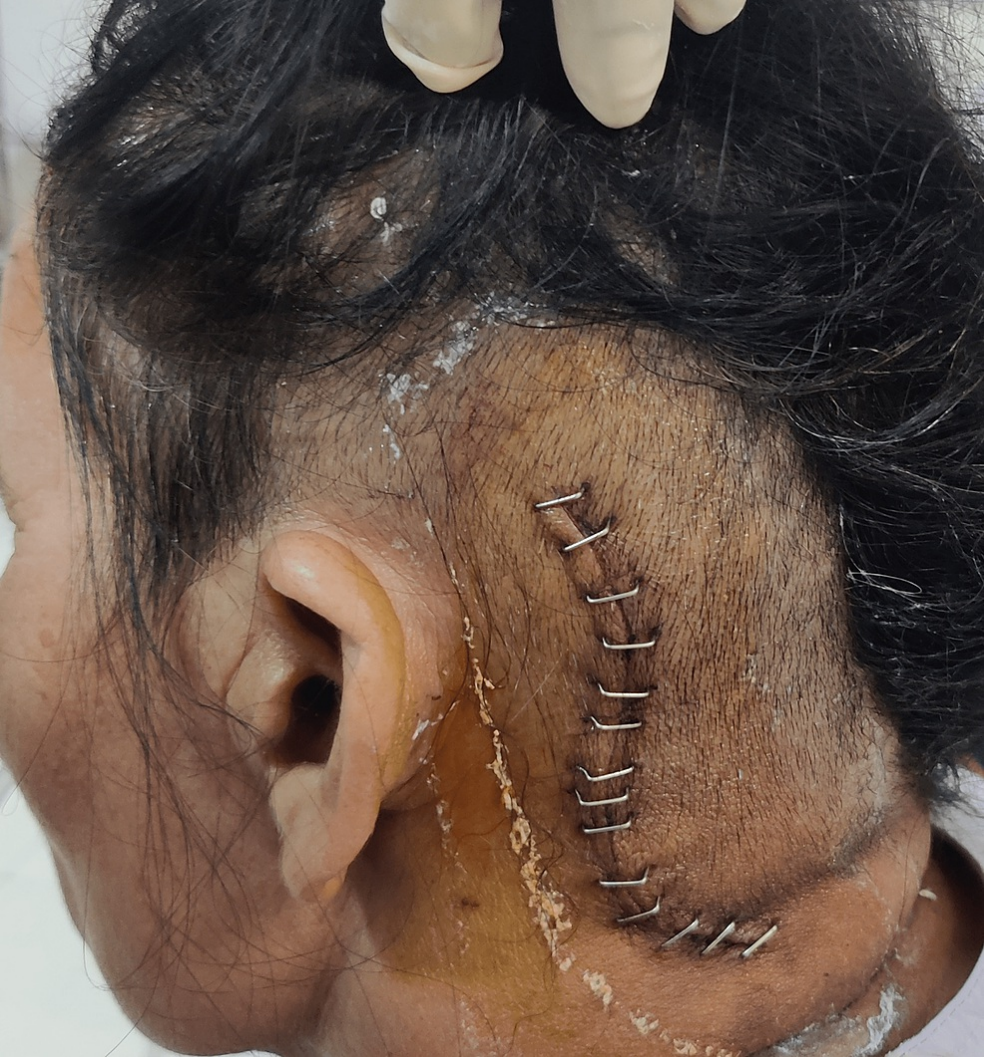
Sutures present over the temporal region (left).

Prior to the examination, proper consent from the patient was obtained. The patient was examined in a sitting and standing position. The patient’s Glasgow coma scale (GCS) score was 15 and she was well-oriented and conscious. On observation, her body build was mesomorphic. On neurological examination, the tone was graded 0 which signifies normal according to the modified Ashworth scale (MAS), and upper, and lower limb range of motion (ROM) and manual muscle testing (MMT) was evaluated which was found to be normal. Cranial nerve examination was done from the first to the 12th cranial nerve and all were intact except the seventh (facial nerve), and eighth (vestibulocochlear) cranial nerve (left). In a sensory examination, superficial, deep, and cortical sensations were evaluated and all were intact. Deep tendon reflexes were also intact. Facial muscles were assessed, and the actions of those muscles were found to be diminished. Table 1 and Figure 1 show facial muscles assessment. On surgical incision, the scar was present on the temporal region of the left side and it was of adherent type. The total number of sutures present was 14 and it is shown in Figure 2.

**Table 1:** Facial muscles assessment.

Facial muscles	Right	Left
Frowning of forehead	Intact	Diminished
Frowning of eyebrows	Intact	Diminished
Orbicularis oculi (opening and closing of eyelids)	Intact	Diminished
Nasal flaring	Intact	Diminished
Puffing of cheeks	Intact	Diminished
Smiling	Intact	Diminished

Coordination tests

The non-equilibrium test was performed and those are stated in Table 2. Romberg’s test was performed in the equilibrium test and was positive in both open and closed eyes. Whenever the patient was initiating walking, she used to feel dizzy. Her static, as well as dynamic balance, was impaired.

**Table 2:** Non-equilibrium tests.

Non-equilibrium test		
Test	Right	Left
Finger to nose	2	2
Finger to finger	2	2
Heel to shin	3	3
Pronation supination	3	3

In the non-equilibrium test, grade 2 indicates moderate impairment and grade 3 indicates minimal impairment.

Timeline 

The date of admission of the patient to the date of follow-up is given in Table 3.

**Table 3:** Timeline of the patient.

Timeline	Date
Date of admission in the hospital	26/10/2022
Date of investigation	27/10/2022
Date of surgery	29/10/2022
Date of physiotherapy reference	01/11/2022
Date of physiotherapy assessment	01/11/2022
Date of follow up	09/01/2023

Outcome measures

The Berg balance scale (BBS) and motion sensitivity quotient (MSQ) were used to evaluate balance and basic mobility activities, which thereby aided in designing the rehabilitation protocol and achieving the performance objectives. MSQ score before therapy was 48. BBS pre-treatment score was 18.

Therapeutic interventions

Tables 4, 5 give a detailed physiotherapy plan for the patient.

**Table 4:** Vestibular and balance rehabilitation therapy. VOR: vestibulo-ocular reflex

Vestibular and Balance Rehabilitation Therapy.			
Intervention	Rationale	Strategy	Regimen
Head movement exercises.	To reduce symptoms and inculcate vestibular responses.	Sustaining focus on a visible object while rotating the head clockwise and counterclockwise, moving the head right and left, and nodding up and down.	For 1-2 minutes, 3-5 times daily. Progression can be made by increasing the velocity of the movements gradually.
Gaze stabilization exercises	To minimize the symptoms of dizziness.	VOR x1 VOR x2	For 1-2 minutes, 3-5 times daily. Progression can be made by increasing the velocity of the movements gradually.
Habituation exercises	To minimize the symptoms of dizziness.	Choose the movement that makes the patient moderately dizzy, and ask the patient to maintain that position for 30 seconds.	For 3-5 times, 2-3 times daily
Balance Exercises	Standing-related exercises serve to enhance balance when standing.	Standing using a parallel bar as support, spot marches, bedside scooting, Body weight shifting (forward-backward and side to side), partial wall squats, stance on 1 leg, tandem standing, reaching out, Perturbations., Stepping (forward-backward and side).	From weeks 3-6 for 10 repetitions x 2 sets for all. From week 6 onward, the patient progressed by performing these tasks while standing alone in a parallel bar.

**Table 5:** Conventional neuro physiotherapy protocol. PNF: proprioceptive neuromuscular facilitation

Conventional Neuro physiotherapy protocol with rationale, the strategy used, and regimen.			
Intervention	Rationale	Strategy	Regimen
Facial PNF	Helps in stimulating the weakened facial muscles.	This is shown in Figure 3.	10 reps for each, 2-3 times per day.
Facial expression exercises with mirror feedback.	Helps to strengthen the facial muscles.	-Frowning of eyebrows. -Closing of eyes -Nasal flaring -Puffing of cheek -Smiling	10 repetitions for each, 2-3 times per day. The progression was made 15 repetitions.
Bedside sitting	Aids in training sit-to-stand transition and improving sitting balance.	Short sitting: feet supported, hip and knee 90 degrees flexed, trunk neutral, and elbows locked. Weight shifting, exercise for trunk movement, Reach outs, Perturbations, Dynamic quadriceps.	From weeks 0-3 for 10 reps x 2 sets for all. Advancement was made by having the patient implement these exercises on a Swiss ball from week 4 onwards.
Sit-to-stand transfer	For strengthening the muscles of lower limbs.	Strategies include: Hip hiking/pelvic lift with holds. Butt walking/scooting. Sit to stand with holds.	From week 3 onwards 10 repetitions 2 sets for all.
Coordination exercises.	To enhance coordination.	Finger to nose Finger to therapist's finger Supination pronation Mass grasp Heel on shin Drawing a circle or figure eight Frenkel exercise in supine lying and sitting position.	10 repetitions x 1 set. Progression was made with respect to speed, intensity, and repetitions.
Ambulation	Aids in normalizing gait patterns and makes walking easier.	Walking (forward-backward and side). -Step up and step down. -Obstacle walking -Gait parameters were emphasized throughout gait training.	Gait Training with assistance in parallel bars from weeks 6-8 and without assistance from week 8 onwards.

Gaze Stabilization Exercises

Vestibulo-ocular reflex (VOR) x1: In order to keep the target in focus, patients were told to maintain their focus on a stationary object. are instructed to keep their eyes fixed on a stationary target positioned in front of themselves while moving their heads for a total of one to two minutes in both horizontal and then in vertical directions.

VOR x2: As the practice advances, more visually interesting targets-like checkerboard, and moving objects are used in which the object moves in the opposite direction to the head motion.

**Figure 3:**
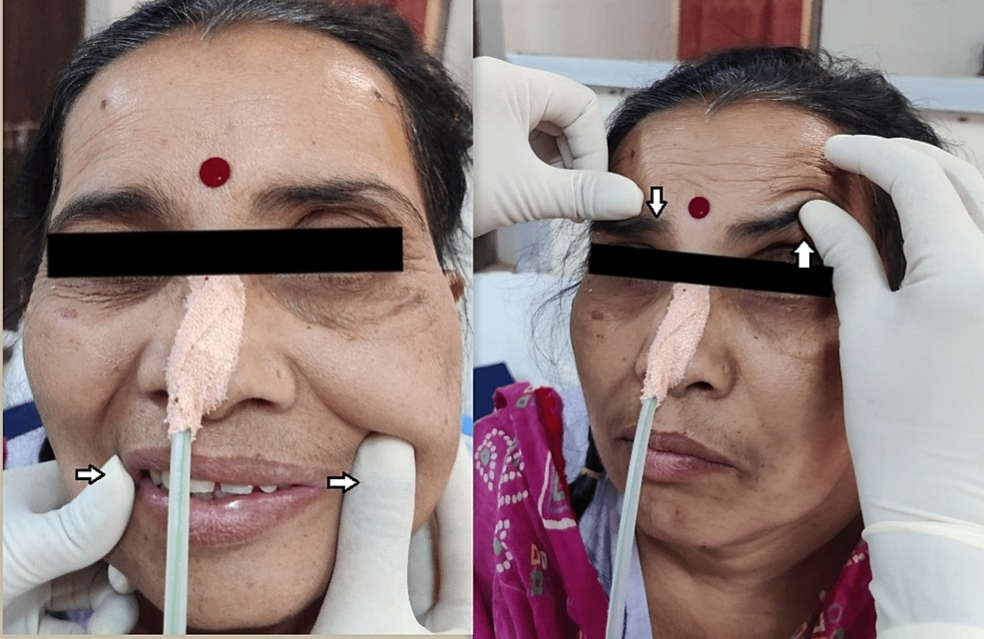
Facial proprioceptive neuromuscular facilitation (PNF).

Follow-up and outcomes

After receiving physical therapy for nine weeks, there was a noticeable improvement. She was able to walk with minimal assistance. Table 6 states the comparison of the scores of outcome measures taken in an interval of a few weeks. After the physiotherapy protocol, the patient has shown a significant increase in his gait and balance. His coordination was also improved, as proper balance was achieved.

**Table 6:** Comparison of outcome measures taken.

Comparison of outcome measures taken			
Scale	0-3 weeks (Score)	3-6 weeks (Score)	6-9 weeks (Score)
MSQ	48	26	07
BBS	18	34	50

MSQ

This shows that initially in 0 to three weeks the score was 48. This means severe symptoms were noticed. In three to six weeks the score was 26. After nine weeks, the patient’s score was 07 which means mild symptoms were seen.

BBS

The score in 0 to three weeks (18) indicates high fall risk, in three to six weeks (34) indicates a moderate risk of falls, and in six to nine weeks (50) indicates a low risk of falls.

## Discussion

The patient was having balance issues, giddiness, and facial muscle weakness. Rehabilitation protocol included many aspects like vestibular and balance rehabilitation therapy, strengthening exercises, facilitating the patient from sitting to standing and walking, etc.

In the study done by Herdman et al., the findings indicated that vestibular adaptation exercises enhance postural stability and reduce the feeling of dizziness in acoustic neuroma patients [9]. Even in the study done by Enticott et al., they concluded that simply VOR exercises and counseling help patients recover from surgery more quickly [10]. Vereeck et al. found that in people over 50 years of age, following an acoustic neuroma surgical procedure, early vestibular therapy helps to regain and shows improvement in motor stability earlier in six weeks of the protocol [6]. According to this prospective clinical trial of Cakrt et al., performing certain exercises with visual cues might help patients recover more quickly from acoustic neuroma surgery by improving their vestibulospinal compensation [4].

A study done by Patarapak et al. demonstrated that when used to treat vertigo and loss of balance caused by several forms of vestibular disorders, the Chulalongkorn vestibular balance therapy had very positive outcomes [11].

The most frequent reason for facial nerve rehabilitation is facial nerve dysfunction brought on by the removal of a vestibular schwannoma. A study done by Rudman et al., says that physiotherapy and biofeedback share an important role in facial movement retraining and synkinesis treatment. The mirror and electromyographic (EMG) feedback are frequently employed to give patients feedback so they may recognize and then lessen synkinesis contractions or make substantial facial expressions [12].

A review by Lindsay et al. says that the facial grading scale (FGS) score enhancements demonstrated that patients may successfully control symptoms with therapy and emphasized the need for specialist therapies in the treatment of facial paralysis [13].

## Conclusions

For patients with disorders similar to cerebellopontine angle (CPA) tumors, a multidisciplinary approach is necessary for a better prognosis. Patient with these conditions has a special possibility to be treated through physical therapy. The results of the motion sensitivity quotient and the Berg balance scale have been shown to be more helpful in reaching performance goals. Once the patient found the right balance, she just needed a little help (<25% assistance or supervision) to carry out her regular tasks. The patient's coordination was also proven to be normal as their balance improved. This enhanced her quality of life as well.
